# Letter from a Clergyman

**Published:** 1854-05

**Authors:** 


### LETTER FROM A CLERGYMAN.

"Springfield, March 21, 1854.

"Dear Sir,—I received your request to communicate, for the public, in your Journal, some account of the *measures I have pursued* to preserve such an amount of health, as the facts stated in my forty-fifth anniversary sermon indicate. You impose on me rather a delicate task, since I must say much of myself, if I say anything. I have always had an aversion to appear before the public, in a formal manner, and therefore have declined applications for sermons to be published. But if my experience will promote the interest or happiness of others, (especially of young men who design to preach the blessed Gospel,) I ought not to decline 'serving my generation.' What I stated was strictly correct. I have not been detained from the sanctuary but one whole day and two half days, by indisposition, during my ministry ; the entire day's detention was not from sickness, but from a dangerous fall down a flight of stairs, which rendered it inconvenient for me to stand in the pulpit. I have not seen a day for more than fifty years, in which I might not have gone abroad, if it had been necessary; no sickness has ever prevented me. As to the measures I have pursued, there has been nothing out of the common course. From early youth I have been strictly abstemious as to all *strong drink.* I have no recollection of ever having mixed a portion of alcohol for beverage. I never drank a glass of wine until I left College, at the age of 21 years. After that time I occasionally drank a glass when I mingled in the social circle, where it was temperately used. At no festival that I ever attended did I drink more than two glasses in the two or more hours of its continuance. I never had a *fondness* for wine, and have long since abandoned the use of it even at *weddings.* During my entire life I have never used stimulating drinks, so as to feel exhilarated by them in the slightest degree. I have never made use of tobacco in any of the forms in which many use it. I was settled, before I was 25, over one of the largest parishes in Massachusetts, and for a period of seven years I was the only minister of any denomination in the town, and was often called on to attend funerals in another small parish, about six miles distant. My own parish was spread over a territory of about seven miles square, and after about six years from my ordination, rapidly increased in population. I usually attended three religious meetings during the week, and preached twice on the Sabbath, and held a prayer and conference meeting in the evening. In one extensive and long continued revival, I attended and conducted a religious meeting every evening in a week, in different parts of my parish, for nine months, when the weather would permit. During my ministry, I have never been obliged to decline, when applied to, to attend a parochial duty. I have visited my people as much as I deemed necessary. In one year I kept an account, and found I had made nine hundred and ninety-six pastoral *calls ;* they could not be called visits, for in most cases I staid but a few minutes, but time enough to drop a word of exhortation, and manifest the interest I felt in the family, and when called on, offered a prayer. I have always used a large amount of exercise, in walking, riding on the saddle, in the first part of my ministry, working in the garden, sawing wood, &c., such exercises as ministers generally use. I have lived as most ministers live; generally on simple food, without many of those condiments which some use freely. I have been an early riser for the principal part of my ministerial life. My sleep has always been *sweet*, like that of the laboring man, and I think on an average would not exceed seven hours. About fifteen years since, in company with two other gentlemen, I walked ten miles in two and a half hours, and stopped nearly ten minutes to rest, and now, at the age of three score and ten, I can walk a single mile in fifteen minutes. I have written not far from twenty-five hundred sermons, many of which I have already committed to the flames, and most of the residue will share the same fate ere long. But alas! my dear brother, I can say with truth, that I have been an unprofitable servant in the vineyard of my blessed master, and I rely only on His righteousness for acceptance on the day of judgment. You may make what use you please of this communication; and publish such extracts as you may think proper. If it shall lead any of my younger brethren to take good care of their health, I shall rejoice. Ministers in these days need good health, for much is required of them.

"I am, dear Sir, your friend and servant,

"SAMUEL OSGOOD."

"W. W. Hall, M. D."

---

MIRTH—is like a flash of lightning that breaks through the gloom of clouds, and glitters for a moment; cheerfulness makes up a kind of daylight in the mind, and fills it with a steady and perpetual serenity.—*Addison.*

